# Endodontic Treatment for a Mesiodens: A Case Report

**DOI:** 10.7759/cureus.57946

**Published:** 2024-04-10

**Authors:** Navdeep Jethi, Gopinder K Jugpal, Jagmohan S Ghumman

**Affiliations:** 1 Conservative Dentistry and Endodontics, Daswani Dental College and Research Centre, Kota, IND; 2 Dentistry, Guru Nanak Dev Dental College and Research Institute, Sunam, IND; 3 Dentistry, DJ College of Dental Sciences, Modinagar, IND

**Keywords:** supernumerary teeth, pediatric dental trauma, developmental anomalies, extra teeth, endodontic treatment, mesiodens

## Abstract

Mesiodens are common supernumerary teeth that prominently erupt in the midline between the maxillary central incisors. If two or more mesiodens are present, they are termed mesiodentes, indicating the presence of multiple supernumerary teeth in the midline. These often cause aesthetic disharmony in the anterior teeth due to their abnormal position, leading to extraction in most cases and resulting in midline diastema when impacted or partially erupted. This case is uncommon, as the patient expressed a desire to preserve their mesiodens as a distinctive feature, considering them a familial trait worth retaining. The family history of the patient revealed the occurrence of mesiodens in three generations, highlighting a hereditary pattern of supernumerary teeth within the family. The endodontic therapy involving root canal treatment successfully treated the mesiodens, alleviating pain, and preserving them as desired.

## Introduction

Mesiodens are the supernumerary teeth in human dentition, with a prevalence of 0.15% and 1.9% in the human demographics [1]. Mesiodens are a positive family trait transmitted as an autosomal character, with a higher occurrence in the maxillary dentition of males than females [2]. Mesiodens are typically located in the midline and can manifest unilaterally on one side or bilaterally on both sides of the midline [1,2].

Morphologically, they are conical, tuberculate, or molariform in shape, with the conical shape being the most common. These teeth are characterized by short roots and are often impacted and inverted [2].

The erupted mesiodens tooth creates disharmony in the aesthetic alignment of the anterior teeth. Mesiodens can frequently cause midline diastema, a gap between the central incisors, when they are impacted or partially erupted [3]. Mesiodens can serve as a distinctive hallmark of an individual's identity, adding to their uniqueness [4].

Clinical diagnosis in the oral cavity is used for erupted mesiodens. X-rays, cone beam computed tomography (CBCT), or panoramic dental X-rays are employed for diagnosing unerupted or partially erupted mesiodens [1-5]. Various myths have been associated with supernumerary teeth, related to luck and omens [6].

Treatment options for mesiodens include extracting impacted or erupted teeth, orthodontic intervention to align teeth affected by mesiodens, or monitoring without intervention if the mesiodens is asymptomatic [1,2,4,5]. This case report discusses endodontic therapy as the chosen treatment for symptomatic mesiodens due to the patient's desire to preserve the tooth and avoid extraction.

## Case presentation

Patient history

A 23-year-old male reported to the Department of Endodontics with a complaint of pain in the upper front teeth and sensitivity to hot beverages like tea. There was a history of trauma due to a sports injury, caused by being hit by a cricket ball, associated with the left central and lateral incisors six years ago, for which no treatment was rendered.

Family history 

The patient had fully erupted mesiodens teeth in between the central incisors. In his family, besides him, his grandmother and his aunt also have a mesiodens tooth. None of the family had ever consulted an orthodontist for malocclusion.

Intraoral examination

**Figure 1:**
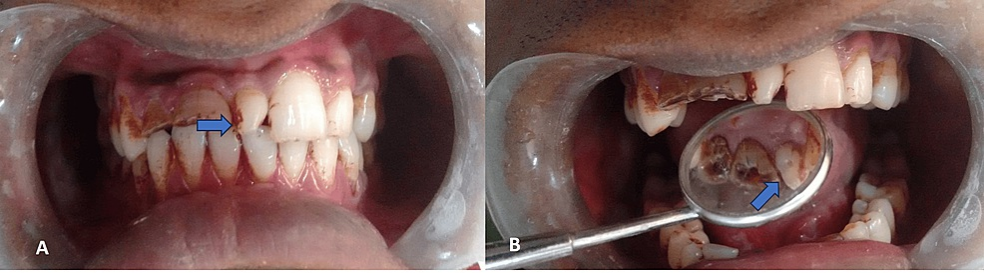
Mesiodens: A) labial aspect, B) lingual aspect

During the intraoral examination, an Elis class III fracture for teeth numbers 11 and 12 was observed. Both teeth were discolored, and there was plaque accumulation and tobacco stains over the teeth. A mesiodens tooth was present between the central incisors. This extra tooth was conical in shape and tender to vertical percussion. Vitality testing with an electric pulp tester indicated that teeth numbers 11 and 12 were nonvital while the mesiodens tooth was vital. The dull pain was associated with teeth 11 and 12, but a sharp throbbing pain was associated with the mesiodens tooth (Figures 1A, 1B).

Radiographic interpretations 

**Figure 2:**
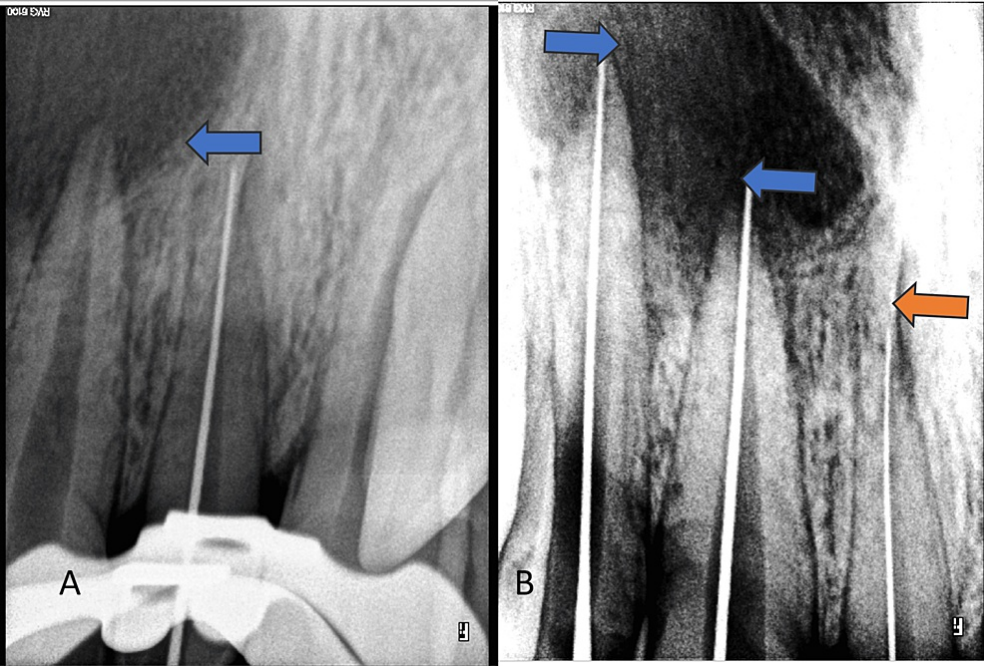
Periapical radiolucency associated with the left central incisor and lateral incisor (blue arrows); no lesion at the apex of the mesiodens (orange arrow)

A periapical radiolucency was associated with teeth 11 and 12. No periapical radiolucency was seen at the apex of the mesiodens tooth. This mesiodens tooth had a wide single canal, a conical crown, and a short root (Figure 2).

Treatment

The treatment plan involved multiple visits for root canal treatment on teeth 11 and 12 and a single visit for endodontic treatment on the mesiodens tooth.

**Figure 3:**
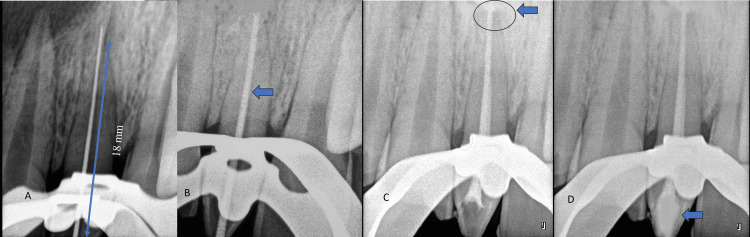
Endodontic treatment: A) working length (18 mm), B) master cone, C) obturation and sealer-puff (blue arrow), D) and post-endo restoration

After an infraorbital and nasopalatine nerve block for the left side was administered with xylocaine, rubber dam isolation was obtained. The access opening for mesiodens involved a ditch in the middle of the cingulum area (Figure 1B) with a number 2 round bur at a 45-degree angle before entering the pulp chamber. A 15k file was used for the initial exploration of the canal. Pulp was extruded, and a glide path was obtained using 10k and 15k files. The lubricant used was ethylene diamine tetraacetic acid (EDTA), and a combination of normal saline and sodium hypochlorite was used as an irrigation solution. Working length was taken with the apex located and digital radiographs, which were 18 mm. Protaper gold files were used for the biomechanical shaping of the canals up to size F3. The obturation was done using gutta-percha size F3 and AH Plus Sealer (Dentsply Sirona, Charlotte, North Carolina, US) using warm condensation with an obturation pen. Post-endo restoration was done using composite resins (Figures 3A-3C).

Anti-inflammatory drugs (Dentogesic DT) were prescribed, to be taken eight hourly in cases of pain. The patient reported no pain within 24 hours after the procedure, during a follow-up telephone call. After the initial visit, the patient did not return for a second visit.

## Discussion

In contrast to previous studies, the mesiodens were retained in this case, as the patient opted to preserve this unique feature of their dentition, highlighting the importance of individual aesthetic preferences and personal choice [4]. Various myths surround supernumerary teeth, with some considering individuals with these extra teeth to be lucky [6], such as the belief in some cultures that supernumerary teeth bring good fortune or represent a connection to the spiritual world.

The patient in this case mentioned his aunt and grandmother having a mesiodens tooth like him. In a study by Alarcón J. et al. (2022), three cases were examined using CBCT to confirm the presence of inherited mesiodens, highlighting the hereditary nature of this dental anomaly [7]. Mesiodens were present for three generations in this case study, involving a grandmother, her daughter, and her grandson, highlighting the strong familial inheritance of genetic factors associated with mesiodens [7].

During tooth development, mesiodens form when the tooth bud splits unevenly to create two teeth, a process unique to the hyperactivity of the dental lamina in developmental theory [2]. The explanation widely accepted in the field indicates that the dental lamina becomes hyperactive, stimulating remnants that develop into additional tooth buds, ultimately leading to the formation of mesiodens [2].

Extraction is the primary treatment for mesiodens due to the pathologies and alignment disturbances they cause in the dentition, which can lead to further complications if left untreated such as increased risks of infection, malocclusion, and damage to neighboring teeth [7-9]. Studies have consistently shown that mesiodens can lead to complications such as crowding, spacing issues, and the rotation of adjacent teeth, which can significantly impact dental alignment and function, according to the findings of these studies [1-3,9]. As well as potential complications like root resorption and delayed eruption, making it the preferred choice to address these issues. [1-3,8,9]. Alsuwaida MM (2024), in his case report, extracted fully erupted mesiodens as a preventive measure for malocclusion [8]. Bhatara S et al. (2024) documented a case series where double mesiodens were causing orthodontic problems in patients and were planned for extraction [9].

Yusa K et al. (2023) conducted a study on 121 mesiodens patients to identify radiographic features and recommend the timely diagnosis and management of mesiodens to avoid future complications [10].

Impacted mesiodens is associated with developmental anomalies, such as an abnormal root formation, an altered path of permanent incisor eruption, and the impaction of permanent incisors, leading to complications like root resorption, delayed eruption, and tooth impaction [1,2,8,9]. Mesiodens can lead to severe complications, such as intraoral infections, cyst formation, delayed eruptions of permanent teeth causing significant discomfort, structural damage to the jaw, and prolonged treatment processes, which may involve surgical interventions and long-term management [1-3]. In rare instances, incisors may even erupt on the nasal floor, posing significant risks to oral health, such as severe infections, airway obstructions, and speech difficulties, necessitating immediate and specialized dental interventions, to prevent further complications and ensure proper oral function and aesthetics [11].

In contrast, the mesiodens tooth was fully erupted, and the issues with the adjacent teeth, including non-vitality, a fracture, and a periapical lesion associated with teeth 11 and 12, were not attributed to the mesiodens but rather were the result of childhood trauma in the present case.

Mesiodens have been linked to various syndromes, including Rubinstein-Taybi syndrome, Nance-Horan syndrome, trichorhinophalangeal syndrome, familial adenomatous polyposis, cleidocranial dysplasia type I, and others, each contributing unique characteristics that correlate with the presence of mesiodens [9].

**Table 1:** Indications and contraindications for endodontic treatment in mesiodens are tabulated based on a review of previous literature This table is based on the review of literature discussed in the discussion and properly cited within the table (not published before in tabulated form).

Indications	Contraindications
Asymptomatic mesiodens (radiographically and clinically) [1-4].	It causes developmental disturbances and pathologies like root resorption in adjacent permanent teeth [1-3].
It does not harm adjacent teeth (radiographically and clinically) [1,2].	It causes periapical infections like cysts [11].
No related pathology (radiographically and clinically) [1,2,4].	It causes malocclusion, like spacing, crowding, rotation of the adjacent tooth, and midline diastema [7-9].
	It is impacted or partially erupted [7,11].

Ethically, we cannot extract a tooth without the patient's consent; if he wants to save it, we should consider it while planning his pain relief. Endodontic treatment of mesiodens (Table 1) is relatively rare, with extractions being the preferred treatment option [1], mainly due to the complex anatomic location of mesiodens in the jaw and associated complications [7-10]. Furthermore, mesiodens can serve as a distinctive identifier for an individual, facilitating recognition even in challenging situations like fire accidents, where unique dental features play a vital role in accurate identification and prompt medical treatment [4]. It is crucial to emphasize that while these teeth can be left undisturbed when asymptomatic [1,2,4,5], they require regular and timely monitoring to ensure the early detection of any potential issues and prompt intervention if necessary [7-9].

## Conclusions

When deciding whether to save a mesiodens tooth, it is crucial to consider both individual preferences and the severity of associated disturbances and pathologies. The family history of mesiodens provides valuable genetic information for the early diagnosis of malocclusion. A fully erupted mesiodens tooth can be retained if it is asymptomatic, free from infections, and shows no complications or pathologies in radiography. Furthermore, fully erupted mesiodens can play a vital role in forensic identifications after fire accidents, facilitating the rapid recognition of individuals by forensic experts.
